# The Role of Personal Values and Student Achievement in Academic Dishonesty

**DOI:** 10.3389/fpsyg.2019.01887

**Published:** 2019-08-16

**Authors:** Maciej Koscielniak, Agnieszka Bojanowska

**Affiliations:** ^1^Faculty in Poznan, SWPS University of Social Sciences and Humanities, Poznań, Poland; ^2^Department of Psychology, SWPS University of Social Sciences and Humanities, Warsaw, Poland

**Keywords:** academic dishonesty, cheating, personal values, grades, motivation

## Abstract

Although multiple individual and environmental causes of students’ dishonest behaviors have been well documented in past research, not much attention has been paid to the human values perspective yet. The current study investigates the direct relationship of values with academic dishonesty, as well as the moderating role of students’ past achievements (grades). Analyses were performed on 219 Polish university students (*M* = 46, *F* = 173). Questionnaire measures were used, including Schwartz’s Portrait of Values Questionnaire. It was found that socially orientated human values (Conformity and Tradition) were negatively related to unethical behaviors, while personally focused values (Hedonism, Power, and Stimulation) correlate positively. Additional analyses revealed that the relationships of some values (Achievement and Security) with academic dishonesty are significantly moderated by students’ academic performance (grades). In the discussion we suggest that academic dishonesty is a pattern of behavior that can be successfully investigated from the perspective of human values – in order to identify its correlates and to plan preventive actions.

## Introduction

The prevalence of academic dishonesty is well documented in most types of educational settings all over the world, with its severe consequences for the functioning of institutions and for the moral development of learners. Moreover, this “cheating crisis” seems to be on the rise (Jones, 2011) and is proven to be a significant predictor of future (e.g., professional) deviant behavior among graduates (Harding et al., 2004). In the current study we address this problem by investigating the links between student cheating and personal values as described in the Theory of Basic Human Values by Schwartz (1992). Attention is also given to students’ past achievement (grades), often considered as the moderator in the values–dishonesty relationship.

Many researchers of academic dishonesty have searched for its causal factors in personality traits (Orzeck and Lung, 2005; Nathanson et al., 2006; Giluk and Postlethwaite, 2015) – assuming that some students may be more likely to engage in misconduct. However, such an approach is somewhat disputable: personality traits are considered endogenous characteristics (Olver and Mooradian, 2003), while academic fraud has also been proven to have a social and environmental basis (McCabe et al., 2001; Murdock et al., 2007; Rettinger and Kramer, 2009). We believe that the perspective of personal values, understood as learned adaptations influenced by the environment (Olver and Mooradian, 2003), offers an interesting opportunity for integrating the personal and situational causes of student dishonesty.

In the existing literature, little research has focused on the relationships between values and academic cheating. Values are presumed to have an impact on numerous patterns of judgment and behavior (Schwartz, 2003), including the ethical dimension of decision making (Fritzsche and Oz, 2007). Feldman et al. (2015) show in their multi-national series of studies that there is a strong and consistent relationship between values and unethical behavior. More specifically, unethical behavior seems to be positively associated with self-enhancement values and negatively associated with self-transcendence and conservation. These results are a further incentive to look for the antecedents of today’s epidemic of academic dishonesty in the domain of human values.

According to common stereotypes, academic cheating during examinations is strongly related to students’ low grades and poor achievements. It is often treated as a form of compensation and a strategy that helps students to achieve satisfactory results via an “easier” path, as suggested by in his review article Cizek (1999). Also, in her study Jones (2011) reveals that grades are the most important reason for cheating in students’ declarations. In the current study, we plan to include students’ past performance (grade point average) as a potential moderator in the relationship of values and academic dishonesty.

We expect that the confirmation of hypotheses about the relationships of values and achievements with academic dishonesty will shed some more light on individual differences in academic cheating, as well as emphasize the role of students’ performance in their attitudes toward fraudulent practices.

### Human Values

Values can be defined as the way people perceive what is important in their life (Schwartz, 2012). They refer to desirable goals and are a guiding system for all kinds of human behavior. According to Schwartz values are structured in a similar (circular) way across different cultures, serving as universal standards and criteria of what is wrong and what is right. Values that are close to one another may be realized at the same time (e.g., Stimulation and Hedonism), because their underlying motivations are similar, while values that lie on the opposite parts of the circle contradict one another: they motivate different kinds of behavior and cannot be realized by one activity (e.g., Benevolence versus Power). Consequently, values that lie closer to one another correlate with other constructs in a similar way, while opposite values tend to have inverse correlations. The circular structure of values also allows for their division into higher order values: values can be grouped into serving individual interests (self-direction, stimulation, hedonism, power, achievement) or collective interests (e.g., conformity, tradition, benevolence, and universalism).

Studies have consistently shown that inclination toward various fraudulent behaviors depends strongly on the structure of one’s values (Fritzsche and Oz, 2007). Altruistic values are likely to make people act ethically, while focusing on self-enhancement is negatively correlated with ethics in decision making. It has been suggested that power and achievement values are related to less socially adaptive behavior and to instrumental use of others (Kasser et al., 2007), while ethical behaviors, such as fair-trade consumer decisions, are predicted by values of universalism and self-direction (Doran, 2009). This last relationship suggests that in some contexts, individualistic values can lead to ethical behavior, so generalizations from one domain onto another must be made with caution, as various moderators of these relationships may come into play.

The relation between ethical behavior and values has also been examined in the area of academic dishonesty: previous research suggested strong and negative correlation between focusing on self-transcendence value and willingness to cheat (Morris, 2012). It is consistent with the results of the series of studies by Feldman et al. (2015): both of these papers portray people’s low levels of Benevolence and Universalism as the root causes of unethical behavior.

We will further investigate the relationship of values and academic cheating inclinations. Our intention is to examine those correlations for all (10) of the values proposed by Schwartz (2012), in order to identify specific functions of each value for cheating behaviors.

Based on the assumption of Schwartz’s model, that opposite values compete against one another in terms of goal realization, we hypothesize that students’ inclination to dishonest behaviors will be positively related to *Personal Focus* values (e.g., Power or Hedonism) and negatively to *Social Focus* values (e.g., Tradition, understood as following customs and being humble; and Conformity, understood as obeying rules and social norms). This has been suggested by previous studies, which indicate that seeking power and achievement may happen at the cost of abiding by social constraints (Kasser et al., 2007), such as the “no-cheating” rule. Contrastingly, social focus values may tend to motivate people to follow these rules, and consequently to avoid cheating behavior.

Values are relatively stable and they tend to serve as consistent guiding systems for behavior. However, their functions can also be mildly modified depending on the context and perception of the current situation. This is crucial for hypothesizing that despite the observed value stability, values can also be used in a different manner by individuals with different needs and motivations. Consequently, it may be vital to distinguish the value–dishonesty analysis into low-achievers and high-achievers, who may be characterized by completely different motivations in their learning strategies. We therefore expect that some of the relationships of values with dishonest inclinations will be moderated by students’ previous achievements (grades). High Security should be a negative predictor of academic dishonesty, especially among low-achievers – whose motivation to avoid future failures will be accompanied by their reluctance to engage in unsafe and risky behaviors. On the other hand, Self-Enhancement values (e.g., Achievement) should especially motivate high-achievers to avoid cheating and aim at mastery in terms of knowledge and competences.

## Materials and Methods

### Subjects

Two hundred and nineteen university students (46 males and 173 females) aged 18–35 years (*M* = 23.27; *SD* = 3.34) participated in this study. 22.8% of them were first year, 18.7% were second year, 25.1% third year, 16.0% fourth year, 16.0% fifth year, and 1.4% sixth year students.

All of them were recruited via a public research panel (SW Research) from different Polish universities throughout the country. They submitted their answers to a Web survey hosted by the research panel. The reward provided for participation were “social points” granted by the research panel (collection of a certain amount of these points entitled participants to receive a small material prize, such as a mug or T-shirt).

The analyzed sample did not include 18 participants (15 females and 3 males; age: *M* = 22.67, *SD* = 3.97) who were identified as outliers due to unrealistic times of study accomplishment or missing answers to key questions (exclusion criteria had been defined before data collection). Data were acquired within seven consecutive days (in June 2015) and analyzed in IBM SPSS software, version 24.

### Measures

#### Academic Dishonesty

Participating students were asked to indicate how likely it would be that they behave in ways that are generally understood as academically dishonest. A brief scenario was presented, describing the situation of a difficult and very important academic examination, for which the participants were unprepared. Five statements were given, describing popular, unethical ways of behaving in this situation (e.g., preparing a cheat sheet or looking at a fellow student’s paper), and students were required to mark the extent to which they would consider such solutions. Answers were assessed on a 7-point Likert scale ranging from “Definitely not” to “Definitely yes.”

The questionnaire was adopted from a survey conducted on a national sample of Polish students (*N* = 514) by Bielska and Hoffman (2013). From among many items used in this study we decided to focus on dishonest behaviors during examinations, excluding other types of dishonesty (e.g., plagiarism). The internal reliability indicators in this report were not included, as the conclusions were discussed mostly in a qualitative manner. The full list of adopted items with English translations is available in the Supplementary Material. Cronbach’s alpha of the scale used in the current study was 0.73, which we consider satisfactory in scientific research.

#### Human Values

To assess human values according to Schwartz’s 10-value model, we used the abbreviated version of the Portrait of Values Questionnaire (PVQ; Schwartz, 2003). This tool is broadly used in the European Social Survey and has been confirmed to show high adequacy for assessing relationships among values, attitudes, behavior, and socio-demographic variables (Davidov et al., 2008).

The questionnaire consists of 21 items, “portraits” of different people. These sentences describe a person’s goals and aspirations with words as “it is important to him/her …” or “he/she thinks,” “he/she believes,” etc. For each of these statements, respondents answered the question: “How much like you is this person?” and checked one of boxes labeled from “Very much like me” to “Not like me at all” (a six-point categorical scale).

In the process of statistical data analysis, the obtained raw scores were ipsatized (centered) to remove the effect of individual differences in mean response level (Schwartz, 2003) and to minimize the response bias and social desirability bias (Cheung, 2016). All human values proposed in this model were determined by two items in the questionnaire (apart from Universalism: consisting of three items as the most complex construct among all values).

We used the Polish version of the PVQ questionnaire, phrased for both male and female respondents (the “/” symbol was used, for example: “It is important to him/her …”). The translations used in the European Social Survey were used and the original order of questions was unchanged (as suggested by Schwartz, 2003).

#### Students’ Achievement Level

All participants were asked to give their approximate grade point average for the previous academic year (first year students gave their grade point average for the last year of secondary school). The best grade given was 5.0 (the maximum score in the Polish grading system) and the worst grade was 3.0 (the lowest grade in Poland is 2.0, but it is not a passing grade).

## Results

### Correlation of Values With Academic Dishonesty

**FIGURE 1:**
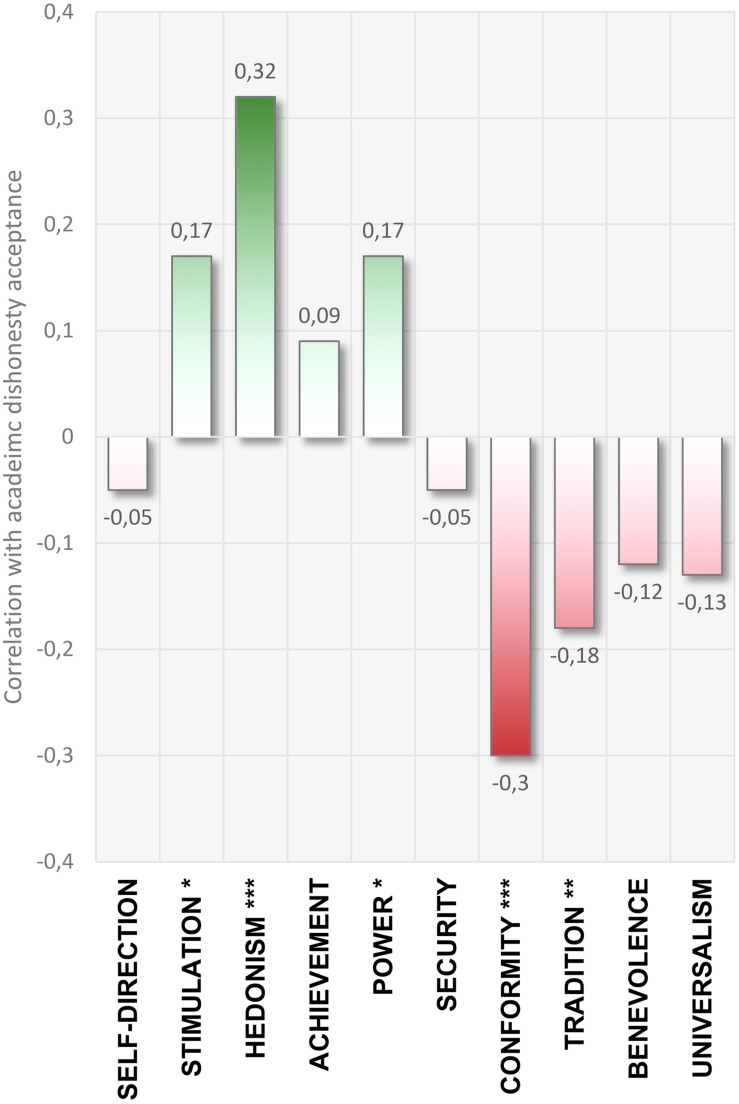
Pearson correlation coefficients between: participants’ inclination toward academic dishonesty and their personal values (according to the theory by Schwartz, 1992). Self-direction, Stimulation, Hedonism, Achievement, and Power are considered Personal-Focus values, while Conformity, Tradition, Benevolence, and Universalism are called Social-Focus values. The extended matrix of the correlation values between all variables used in this study is included in the Supplementary Materials. ^∗^*p* < 0.05; ^∗∗^*p* < 0.01; ^∗∗∗^*p* < 0.001.

To examine the general relationships of values with academic dishonesty, all the PVQ scores were correlated with the score on the Academic Dishonesty Scale. Pearson correlation coefficients revealed that some of the values were significantly and positively correlated with cheating tendencies (Stimulation, Hedonism, and Power), while some others correlate negatively (Conformity and Tradition). The values of Pearson correlation coefficients are presented in Figure 1, while the complete correlation matrix of all variables used in the study is included in the Supplementary Materials.

### Correlation of Grade Point Average With Academic Dishonesty

Pearson correlation coefficients were also calculated to test the overall relationship of academic grades with their scores on the Academic Dishonesty Scale. The result of this analysis indicated that there was a significant, albeit weak, negative association between students’ grade point averages and their inclinations toward academically dishonest practices, *r*(219) = −0,22; *p* = 0.001.

### Moderation Analyses

**FIGURE 2:**
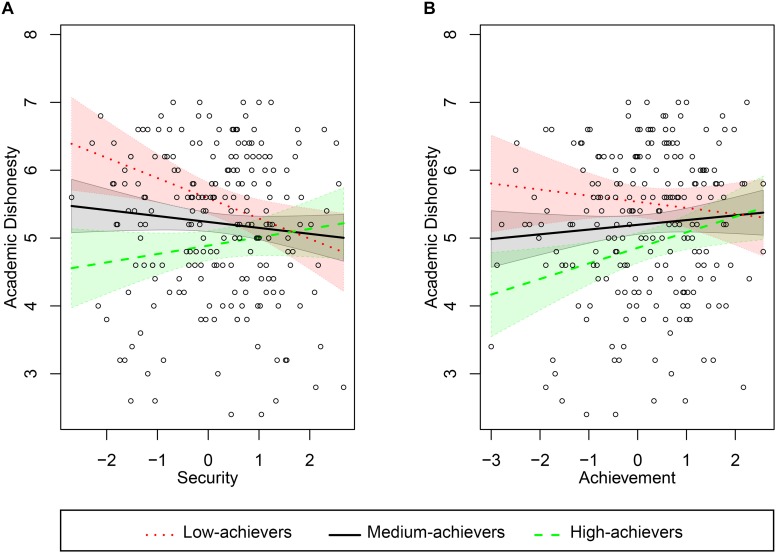
Relationships of personal values (**A**, security; **B**, achievement) with students’ tendency to engage in academic dishonesty, moderated by the level of previous academic achievement (grades). The compared groups of observations refer to the grade point average declared by the participants: estimated values of moderator correspond to the 16th percentile (3.5; *N* = 67), the median grades (4.0, *N* = 79), and the 84th percentile (4.5, *N* = 74).

Analyses of the moderating character of students’ previous achievement on the relationship of their values with cheating tendencies were conducted using PROCESS software (Hayes, 2012). PROCESS model 1 was chosen, as suggested by the author, for estimating, testing, and probing interactions in ordinary least squares regression. In these analyses we used the Academic Dishonesty Scale score as an examined (dependent) variable and low-level value scores as predictors (independent variables). All the interactions of students’ grades with their values were investigated, yielding two statistically significant moderations (Figure 2).

The bootstrapping method (*n* = 10,000 bootstrap resamples, 95% CI) revealed that Security is significantly and negatively related to one’s acceptance of academic dishonesty, but only in the group of low achievers (the unstandardized beta-coefficient between the predictor and the dependent variable is −0.30; *p* = 0.008). Among medium achievers this relationship is weaker and insignificant (β = −0.09; *p* = 0.177), while among high achievers the beta-coefficient is positive (0.12) and insignificant (*p* = 0.201). The *R*^2^ change due to interaction is significant: Δ*R*^2^ = 0.03; *F*(1,215) = 6.68; *p* = 0.010 – indicating the effect of moderation beyond the main effects of independent and moderating variables (Figure 2A).

The relationship of Achievement with scores on the Academic Dishonesty Scale varies as a function of students’ achievement levels: for low-achievers it is negative and insignificant (β = −0.09; *p* = 0.410), for medium achievers it is positive and insignificant (β = 0.07; *p* = 0.270), and for high-achievers it is positive and significant (β = 0.23; *p* = 0.020). The moderator’s effect on *R*^2^ change is significant: Δ*R*^2^ = 0.02; *F*(1,215) = 4.22; *p* = 0.041 (Figure 2B).

## Discussion

As predicted, Schwartz’s human values theory provides a framework within which to investigate the role of values in relation to students acting dishonestly. We confirmed that Personal Focus values are positively correlated with susceptibility to cheat, while in the case of Social Focus values, this relationship is opposite. The pattern of these relationships is fully consistent with the logic of Schwartz’s circular model: values located in the opposite regions of the measurement space tend to show the opposite sign of Pearson’s correlation with academic dishonesty. This outcome questions one of the popular theories of academic dishonesty: according to which we live in the culture of increasing dishonesty – and cheating is the result of social conformism (*collaborative dishonesty*, McCabe et al., 2001). We demonstrate that in the academic environment, a social focus may inhibit the cheating tendencies, while being focused on one’s own interest can boost students’ dishonesty.

We confirmed the social stereotype about negative relationship of students’ grades with their willingness to cheat (although this correlation is quite weak). The group that is most affected by temptation to cheat are underperforming students with low Security values (limited need for safety, low feeling of harmony, and stability of self). Cheating is a risky behavior that threatens the perception of self (as is the case most unethical practices) so it is not surprising that low Security boosts existing cheating tendencies among low-achieving students. On the other hand, high performing students with low Achievement goals are least likely to cheat, as they do not experience the need to prove anything to others and they learn just for themselves.

The results of this study are also complementary to the conclusions of Morris (2012), who assumed a direct relationship between academic dishonesty and a low level of self-transcendent values (Universalism and Benevolence). Although these correlations were not fully confirmed in our study (they are negative, but insignificant), we demonstrated that Self-Enhancement values (opposite to Self-Transcendence in Schwartz’s circular model) are positive predictors of academic cheating. Hedonism and Power strengthen such inclinations overall, while Achievement is related to dishonest behaviors for high achievers (students with the best grades).

Our outcomes can be treated as an inspiration for further analyses of the relationships of personal values with academic dishonesty. Values have a strong impact on human perception, judgments, and behavior; however, they are described as less stable across a lifetime than personality traits (Parks-Leduc et al., 2015). They can be significantly changed by life experiences and social influence (Schwartz, 2011). Confirmation of the causal (not only correlational) relation between values and academic dishonesty in future studies could open new possibilities for preventing dishonest inclinations through careful shaping of socially desired values among children and adolescents. This idea could work even as a short-term intervention, as was suggested by Ariely (2012), who noticed that honor code priming could decrease cheating tendencies among students.

The current study is focused mainly on people’s individual differences (in the fields of values and academic achievements). However, we believe that this is just a part of the “truth” about academic cheating – and in order to get a more comprehensive view, social context should also be taken into consideration, wrote that “one bad apple could spoil the barrel” Ariely (2012), so exposure to many dishonest behaviors may be a good reason to conform. Students realize that they act unethically, but at the same time they are very creative in the rationalization of such choices. This process is called “neutralization” (Sykes and Matza, 1957): being able to perceive one’s own behavior as situationally appropriate is a step toward delinquent acts without experiencing self-esteem damage. Combining this perspective with our concept of human values as predictors of academic dishonesty can be suggested as an interesting idea for further research.

There are several limitations of this study. Its web-based character ensures anonymity and increases the probability of honest answers in the ethically sensitive topic of dishonest activities; however, there are also multiple methodological disadvantages. They include recruitment using non-probability sampling or uncontrolled features related to participants’ context (Skitka and Sargis, 2005). All data were collected in Poland, which renders the results non-generalizable to other countries (especially those with a collectivist culture). Due to the self-reported character of students’ grades, response biases (connected with memory limitations) are possible. The vast majority of participants were female, proven to be less likely to act unethically in Bersoff’s (1999) study. Moreover, the analyses conducted have a correlational character and, thus, we cannot derive any conclusions regarding causal effects. For future research, studies employing structural equation modeling may be useful in the identification of directional effects and should be considered for the continuation of this work.

Due to the nature of the online data collection method we used the short version of Schwartz’s PVQ questionnaire. The unquestionable advantage of this tool is its popularity and presence in the European Social Survey – but some of its shortcomings are also mentioned in the literature (e.g., limited discriminant validity of the different values; Knoppen and Saris, 2009). Moreover, Schwartz’s circular model of values has been intensely developed since the short PVQ tool was created, proving that there can be more values to be distinguished in this model, as many as 19 (Lee et al., 2019). Lastly, Schwartz’s model presents an effective framework for conducting this type of research (it is commonly used and amended), but it is not the only value theory in psychology. It is possible that further evidence on the dishonesty–values relationship can be found in other theories, e.g., by Rokeach (1973) or Hofstede (1980).

Despite all those limitations, the results seem to fit well into the growing trend of exploring psychological underpinnings of academic dishonesty. Such behaviors are often described as the “epidemic problem” (Haines et al., 1986; Barnhardt, 2016) so knowledge about relationships between values and students’ past achievements (grades) can be useful for further research and for planning prevention campaigns.

## Data Availability

The datasets generated for this study are available on request to the corresponding author.

## Ethics Statement

The study was based on a secondary analysis of previously collected data by an external company (Internet agency: SW Research). The data were provided to the authors in an anonymized format. An ethics approval was not required for this study as per applicable institutional and national guidelines and regulations. Brief information regarding the study was provided to all participants prior to procedure initialization, including description of question characteristics and time required to complete the questionnaire. Only accepting this statement by clicking the proper button allowed participants to begin the procedure, and was treated as a receipt of consent.

## Author Contributions

MK designed the study and collected the data. Both authors analyzed the data and wrote the manuscript.

## Conflict of Interest Statement

The authors declare that the research was conducted in the absence of any commercial or financial relationships that could be construed as a potential conflict of interest.
