# Supplementary material for: The Role of Personal Values and Student Achievement in Academic Dishonesty
Source: Front Psychol. 2019 Aug 16;10:1887. doi: 10.3389/fpsyg.2019.01887 (PMC6706799; doi:10.3389/fpsyg.2019.01887)
Supplement: Supplementary file 1 [file Table_1.DOCX]

**SUPPLEMENTARY MATERIALS**

**Questionnaire used in the study to assess students’ willingness to behave dishonestly during examinations:**

| **English version** | **Polish version (used in the study)** |
| --- | --- |
| **INSTRUCTION:**  **Imagine you are writing a challenging exam, important in your curriculum, for which you had difficulty preparing. You know that other people in your group had similar difficulties.**  **Evaluate the probability, that you will behave in the ways described below (think of actual past situations):** | **INSTRUKCJA:**  **Piszesz bardzo trudny i ważny z punktu widzenia Twoich studiów egzamin, do którego nauka przychodziła Ci z wielkim trudem. Wiesz również, że większość osób z Twojego roku miało z tym trudności.**  **Oceń szanse, że zachowasz się jak w podanych niżej przykładach (przypomnij sobie rzeczywiste sytuacje):** |
| 1. Would you prepare materials (cheat sheet) with the most difficult issues and definitions? | 1. Czy przygotujesz sobie pomoc naukową (ściągę) z najtrudniejszymi zagadnieniami i definicjami? |
| 2. It is common knowledge that the instructor used the same exam questions in the previous years. Would you look up the exam copies circulating on campus? | 1. Wiadomo, że prowadzący co roku daje studentom do rozwiązania ten sam arkusz egzaminacyjny. Czy zajrzysz do krążących od lat po wydziale kopii? |
| 3. You are sitting next to the best student from your group and you are able to see his/her answers on his/her answer sheet. Do you use the opportunity to compare your answers with his/hers? | 1. Siedzisz obok najlepszego/j na roku studenta/ki i jesteś w stanie zobaczyć odpowiedzi na jego/jej arkuszu. Czy skorzystasz z okazji porównania swoich odpowiedzi z jego/jej? |
| 4. If the examiner left the room for a couple of minutes, would you use that time to consult your books or notes? | 1. Czy gdyby prowadzący egzamin wyszedł z sali na kilka minut – skorzystałbyś / skorzystałabyś w tym czasie ze swoich notatek lub książek? |
| 5. Your colleague sitting next to you asks you to help him/her with an answer, which you had no difficulty with. Will you help him/her? | 1. Siedzący /a obok Ciebie kolega /koleżanka prosi o pomoc w udzieleniu odpowiedzi na pytanie, które nie sprawiło Ci szczególnego problemu. Czy pomożesz z mu/jej? |
| Each question is to be answered using the following scale  1. Definitely not 2. No 3. Probably not 4. Undecided 5. Probably yes 6. Yes 7. Definitely yes | Dostępne możliwości odpowiedzi na każde z pytań:   1. Zdecydowanie nie 2. Nie 3. Raczej nie 4. Ani tak, ani nie 5. Raczej tak 6. Tak 7. Zdecydowanie tak |

*All of the items were used by Bielska and Hoffman (2013) in the national report on cheating and plagiarism.*

**List of 21 PVQ Items used for assessing students’ personal values (Schwartz, 2003):**

**BENEVOLENCE**

12. It's very important to him to help the people around him. He wants to care for other people.

18. It is important to him to be loyal to his friends. He wants to devote himself to people close to him.

**UNIVERSALISM**

3. He thinks it is important that every person in the world be treated equally. He wants justice for everybody, even for people he doesn’t know.

8. It is important to him to listen to people who are different from him. Even when he disagrees with them, he still wants to understand them.

19. He strongly believes that people should care for nature. Looking after the environment is important to him.

**SELF-DIRECTION**

1. Thinking up new ideas and being creative is important to him. He likes to do things in his own original way.

11. It is important to him to make his own decisions about what he does. He likes to be free to plan and to choose his activities for himself.

**STIMULATION**

6. He likes surprises and is always looking for new things to do. He thinks it is important to do lots of different things in life.

15. He looks for adventures and likes to take risks. He wants to have an exciting life.

**HEDONISM**

10. Having a good time is important to him. He likes to “spoil” himself.

21. He seeks every chance he can to have fun. It is important to him to do things that give him pleasure.

**ACHIEVEMENT**

4. It is very important to him to show his abilities. He wants people to admire what he does.

13. Being very successful is important to him. He likes to impress other people.

**POWER**

2. It is important to him to be rich. He wants to have a lot of money and expensive things.

17. It is important to him to be in charge and tell others what to do. He wants people to do what he says.

**SECURITY**

5. It is important to him to live in secure surroundings. He avoids anything that might endanger his safety.

14. It is very important to him that his country be safe from threats from within and without. He is concerned that social order be protected.

**CONFORMITY**

7. He believes that people should do what they're told. He thinks people should follow rules at all times, even when no-one is watching. 1

6. It is important to him always to behave properly. He wants to avoid doing anything people would say is wrong.

**TRADITION**

9. He thinks it's important not to ask for more than what you have. He believes that people should be satisfied with what they have.

20. Religious belief is important to him. He tries hard to do what his religion requires.


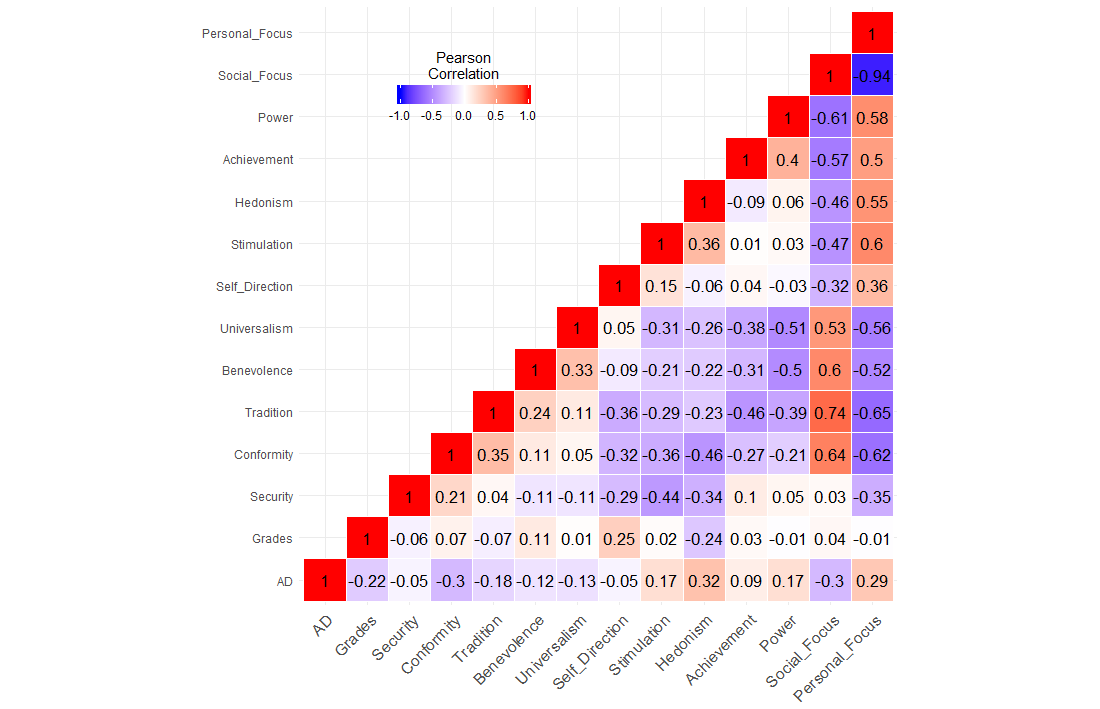


*Figure A.* Pearson’s correlation matrix of variables analysed in the study, illustrating the relationships between academic dishonesty (AD), grades, and human values (of lower and higher order).
